# Inflammation-Driven Lipid Suppression in Hospitalized Patients: Insights Into the Inflammatory Lipid Paradox From a Retrospective Study

**DOI:** 10.7759/cureus.89488

**Published:** 2025-08-06

**Authors:** Kyle E Thurmann, Trisha G Mukherjee, Michael D White

**Affiliations:** 1 Medicine, Creighton University School of Medicine, Phoenix, USA; 2 Cardiology, Creighton University School of Medicine, Omaha, USA

**Keywords:** cardiovascular risk stratification, c-reactive protein, inflammatory lipid paradox, lipid metabolism, systemic inflammation

## Abstract

Introduction

Systemic inflammation alters lipid metabolism by suppressing hepatic lipoprotein synthesis, increasing catabolism, and impairing reverse cholesterol transport. These changes result in reduced levels of low-density lipoprotein (LDL), high-density lipoprotein (HDL), and total cholesterol (TC), despite elevated cardiovascular risk, which is a phenomenon termed the "inflammatory lipid paradox." While well-characterized in chronic inflammatory diseases, such as rheumatoid arthritis, its prevalence and clinical impact in hospitalized adults with systemic inflammation remain underexplored. We investigated whether elevated C-reactive protein (CRP) levels across a range of acute illnesses are associated with progressive reductions in LDL, HDL, and TC, aiming to evaluate the inflammatory lipid paradox as a widespread phenomenon in hospitalized adults.

Methods

We conducted a retrospective analysis of 5,060 hospitalized adults at a tertiary academic center between January 2020 and May 2024. Inclusion criteria were age ≥18 years, CRP >5 mg/L, and availability of a complete lipid panel. Patients were stratified into four CRP categories: 5-20 mg/L, 20-50 mg/L, 50-100 mg/L, and >100 mg/L. Mean LDL, HDL, TC, and triglyceride levels were compared using one-way analysis of variance (ANOVA) with Tukey’s honestly significant difference post-hoc testing. Linear regression, including 95% confidence intervals (CIs) for all regression estimates, was used to assess associations between CRP category and lipid values.

Results

One-way ANOVA revealed that increasing CRP was significantly associated with stepwise reductions in LDL (105.53-87.94 mg/dL), HDL (48.89-38.68 mg/dL), and TC (187.04-157.28 mg/dL) (p < 0.0001 for all comparisons). Triglycerides showed a non-linear trend. Regression analyses demonstrated strong inverse associations between CRP and LDL (slope = -6.09 mg/dL, 95% CI: -10.79 to -1.38, p = 0.0308, R² = 0.94), HDL (slope = -3.51 mg/dL, 95% CI: -4.55 to -2.47, p = 0.0047, R² = 0.99), and TC (slope = -10.30 mg/dL, 95% CI: -17.76 to -2.83, p = 0.0272, R² = 0.95), consistent with inflammation-driven lipid suppression. The slope for triglycerides was positive (4.29 mg/dL), but the association was not statistically significant (95% CI: -15.04 to 23.62, p = 0.4402, R² = 0.31).

Conclusion

Elevated CRP is significantly associated with lower LDL, HDL, and TC levels in hospitalized adults, supporting the presence of the inflammatory lipid paradox beyond chronic disease. These findings highlight the need to interpret lipid panels in the context of systemic inflammation, as suppressed lipid values may both obscure and reflect increased cardiovascular risk driven by cytokine-mediated dysregulation of lipid metabolism. Repeat lipid testing following recovery from acute illness is essential to guide accurate cardiovascular risk stratification and appropriate preventive care.

## Introduction

Systemic inflammation significantly disrupts lipid metabolism by suppressing hepatic lipoprotein synthesis, increasing lipoprotein catabolism, and impairing reverse cholesterol transport. These changes lead to reduced concentrations of low-density lipoprotein (LDL), high-density lipoprotein (HDL), and total cholesterol (TC), with possible elevations in triglycerides (TGs) [1,2]. This constellation of changes, known as the inflammatory lipid paradox, has been described in various inflammatory states, including infection, autoimmune disease, and critical illness.

This paradox presents a clinical challenge: suppressed lipid levels during systemic inflammation may not reflect a patient’s baseline atherogenic risk. Reliance on lipid panels obtained during hospitalization, particularly in the setting of elevated inflammatory markers, such as C-reactive protein (CRP), may lead to underestimation of cardiovascular (CV) disease (CVD) risk and delays in initiating appropriate lipid-lowering therapy [3,4]. Moreover, evidence shows that, in rheumatoid arthritis (RA), lower circulating LDL and TC levels are paradoxically associated with increased CV risk. This may result from inflammation-induced shifts in lipoprotein composition, including smaller LDL particles, dysfunctional HDL, and a pro-atherogenic apolipoprotein cargo, all of which may precede even the clinical onset of RA [5,6].

While the inflammatory lipid paradox is well-documented in chronic inflammatory conditions, especially autoimmune diseases such as RA, its presence and impact in the general inpatient population across a spectrum of acute illnesses remain poorly characterized [7-9]. To address this gap, the present study examines whether CRP elevation, regardless of underlying illness, is associated with reductions in LDL, HDL, and TC levels, with the aim of evaluating the inflammatory lipid paradox as a potentially generalizable phenomenon in hospitalized adults. By stratifying lipid values across elevated CRP categories, we aimed to characterize inflammation-driven lipid suppression during acute illness and assess its implications for CV risk assessment. We hypothesized that increasing CRP levels would be associated with stepwise reductions in LDL, HDL, and TC, consistent with inflammation-mediated dysregulation of lipid metabolism.

## Materials and methods

Study design and data source 

This retrospective study used convenience sampling to analyze de-identified electronic medical record data from a tertiary academic medical center between January 2020 and May 2024. All data were extracted from the hospital’s electronic health record system (Epic Systems Corporation, Verona, WI), with access limited to authorized personnel and storage on secure institutional servers.

Study population and sample size 

Eligible participants were adults aged ≥18 years hospitalized with a documented CRP level >5 mg/L and an accompanying complete lipid panel. To ensure biologic relevance, we restricted inclusion to lipid panels obtained during the same inpatient encounter and within the same year of life as the CRP measurement. Patients were excluded if any component of the lipid panel or the corresponding CRP value was missing within this timeframe; no imputation was performed. For patients with multiple qualifying CRP-lipid pairs, only the earliest encounter was retained to avoid repeated measures. This yielded a final sample of 5,060 unique patients.

Study measures 

A CRP threshold of >5 mg/L was selected to identify clinically meaningful systemic inflammation, rather than low-grade or borderline elevations. Prior studies have shown that CRP levels >3 mg/L are associated with increased CV risk, while levels >5 mg/L more reliably reflect acute-phase responses and inflammation-induced metabolic alterations relevant to lipid suppression [10,11]. We included the full spectrum of elevated CRP values, including those >100 mg/L, to accurately represent the inflammatory burden commonly observed in hospitalized patients. Excluding these higher values could have underestimated the range of metabolic effects in this clinical context [1,12].

Ethics statement 

This study utilized retrospective data and was conducted in compliance with institutional guidelines for secondary use of protected health information. It received institutional review board approval with a waiver of informed consent under an expedited review pathway.

Statistical analysis 

Patients were stratified into four CRP categories: 5-20 mg/L, 20-50 mg/L, 50-100 mg/L, and >100 mg/L. Mean lipid levels were compared across CRP strata using one-way analysis of variance (ANOVA), followed by post-hoc Tukey’s honestly significant difference (HSD) tests for pairwise group comparisons. Linear regression was performed to assess the relationship between CRP category and each lipid component, treating CRP as an ordinal predictor. The coefficient of determination (R²) was calculated for each model to evaluate the strength of association, with R² ≥0.90 indicating a strong model fit. Additionally, 95% confidence intervals (CIs) were computed for all slope estimates.

Sensitivity analyses 

Two sensitivity analyses were conducted. First, patients with extreme values of CRP or lipids were excluded to minimize the influence of outliers. Second, CRP was modeled as a continuous log-transformed variable in linear regression rather than as a categorical predictor. In both models, the inverse associations between CRP and LDL, HDL, and TC remained statistically significant and directionally consistent. TG levels, which followed a non-linear trend in the primary analysis, did not show significant associations in sensitivity testing.

Software and statistical significance 

All analyses were conducted using Python (version 3.13.4), employing the following libraries: pandas for data manipulation, numpy for numerical operations, scipy.stats (linregress) for regression analysis, and matplotlib.pyplot for visualization. A two-sided p-value <0.05 was considered statistically significant.

## Results

A total of 5,060 hospitalized adult patients were included in our analysis, with a mean age of 50.5 years and 53.4% (N = 2,702) identifying as female. The cohort was 58.1% (N = 2,940) Hispanic/Latino patients, 40.9% (N = 2,070) non-Hispanic patients, and 1% (N = 51) unknown, with the following racial distribution: 80.5% (N = 4,073) White patients, 11.3% (N = 572) Black or African American patients, 2.9% (N = 147) American Indian/Alaska Native patients, 0.7% (N = 35) Pacific Islander patients, 1.5% (N = 76) Asian patients, and 3.0% (N = 152) unknown.

Stratification by CRP level revealed a consistent inverse association between systemic inflammation and lipid parameters. Mean LDL decreased from 105.53 mg/dL in the 5-20 mg/L CRP group to 87.94 mg/dL in the >100 mg/L group. HDL declined from 48.89 mg/dL to 38.68 mg/dL, and TC fell from 187.04 mg/dL to 157.28 mg/dL. TGs followed a non-linear pattern, reaching their lowest mean in the 20-50 mg/L group before rising to 186.74 mg/dL in the >100 mg/L group. One-way ANOVA demonstrated statistically significant differences across CRP categories for LDL (F = 68.53, p < 0.0001), HDL (F = 94.83, p < 0.0001), TC (F = 99.39, p < 0.0001), and TGs (F = 5.39, p = 0.0011). These results are summarized in Table 1.

**Table 1:** Mean lipid levels by CRP category in hospitalized patients Mean triglyceride, LDL, HDL, and total cholesterol levels are presented across four CRP categories: 5-20, 20-50, 50-100, and >100 mg/L. One-way ANOVA demonstrated statistically significant inverse associations between CRP and LDL (F = 68.53, p < 0.0001), HDL (F = 94.83, p < 0.0001), and total cholesterol (F = 99.39, p < 0.0001), supporting a lipid-lowering effect of systemic inflammation. CRP, C-reactive protein; LDL, low-density lipoprotein; HDL, high-density lipoprotein; ANOVA, analysis of variance

CRP Category (mg/L)	Triglycerides (mg/dL)	LDL (mg/dL)	HDL (mg/dL)	Total Cholesterol (mg/dL)
5–20	174.31	105.53	48.89	187.04
20–50	163.55	97.66	46.26	174.11
50–100	169.16	89.57	41.78	160.45
>100	186.74	87.94	38.68	157.28
ANOVA F-value	5.39	68.53	94.83	99.39
ANOVA p-value	0.0011	<0.0001	<0.0001	<0.0001

Post-hoc Tukey HSD testing revealed that LDL levels differed significantly across multiple pairwise comparisons: 20-50 vs 50-100 (-8.09 mg/dL), 20-50 vs 5-20 (7.87 mg/dL), 20-50 vs >100 (-9.72 mg/dL), 50-100 vs 5-20 (15.96 mg/dL), and 5-20 vs >100 (-17.60 mg/dL), all p < 0.0001. HDL declined significantly in 20-50 vs 5-20 (2.63 mg/dL, p = 0.0013), 5-20 vs 50-100 (-7.12 mg/dL), 5-20 vs >100 (-10.21 mg/dL), 20-50 vs 50-100 (-4.49 mg/dL), 20-50 vs >100 (−7.58 mg/dL), and 50-100 vs >100 (-3.09 mg/dL), which are all p < 0.01. TC was consistently lower across all group comparisons, particularly for 5-20 vs >100 (-29.77 mg/dL, p < 0.0001). For TGs, significant differences were observed in 20-50 vs >100 (23.19 mg/dL, p = 0.0005) and 50-100 vs >100 (17.58 mg/dL, p = 0.0496). Full pairwise results are presented in Table 2.

**Table 2:** Tukey's HSD post-hoc comparisons of mean lipid levels across CRP categories This table presents pairwise comparisons of mean lipid levels (triglycerides, LDL, HDL, and total cholesterol) across four CRP categories: 5-20, 20-50, 50-100, and >100 mg/L. The first column identifies the lipid parameter. The second column lists the CRP category pairs compared. The third column displays the mean difference in lipid levels (mg/dL), and the fourth column reports the corresponding p-value from Tukey’s HSD test. Statistically significant comparisons (p < 0.05) indicate meaningful differences in lipid levels between CRP strata, supporting inflammation-associated modulation of lipid metabolism. Tukey's HSD, Tukey's honestly significant difference; CRP, C-reactive protein; LDL, low-density lipoprotein; HDL, high-density lipoprotein

Lipid Parameter	CRP Category Comparison (mg/L)	Mean Difference in Lipid Level (mg/dL)	p-value
Triglycerides	20-50 vs 50-100	5.69	0.833
Triglycerides	20-50 vs 5-20	10.76	0.1177
Triglycerides	20-50 vs >100	23.19	0.0005
Triglycerides	50-100 vs 5-20	5.15	0.8208
Triglycerides	50-100 vs >100	17.58	0.0496
Triglycerides	5-20 vs >100	12.43	0.0708
LDL	20-50 vs 50-100	-8.09	<0.0001
LDL	20-50 vs 5-20	7.87	<0.0001
LDL	20-50 vs >100	-9.72	<0.0001
LDL	50-100 vs 5-20	15.96	<0.0001
LDL	50-100 vs >100	-1.63	0.8324
LDL	5-20 vs >100	-17.6	<0.0001
HDL	20-50 vs 50-100	-4.49	<0.0001
HDL	20-50 vs 5-20	2.63	0.0013
HDL	20-50 vs >100	-7.58	<0.0001
HDL	50-100 vs 5-20	7.12	<0.0001
HDL	50-100 vs >100	-3.09	0.0023
HDL	5-20 vs >100	-10.21	<0.0001
Total Cholesterol	20-50 vs 50-100	-13.66	<0.0001
Total Cholesterol	20-50 vs 5-20	12.94	<0.0001
Total Cholesterol	20-50 vs >100	-16.83	<0.0001
Total Cholesterol	50-100 vs 5-20	26.59	<0.0001
Total Cholesterol	50-100 vs >100	-3.17	0.6387
Total Cholesterol	5-20 vs >100	-29.77	<0.0001

**Figure 1:**
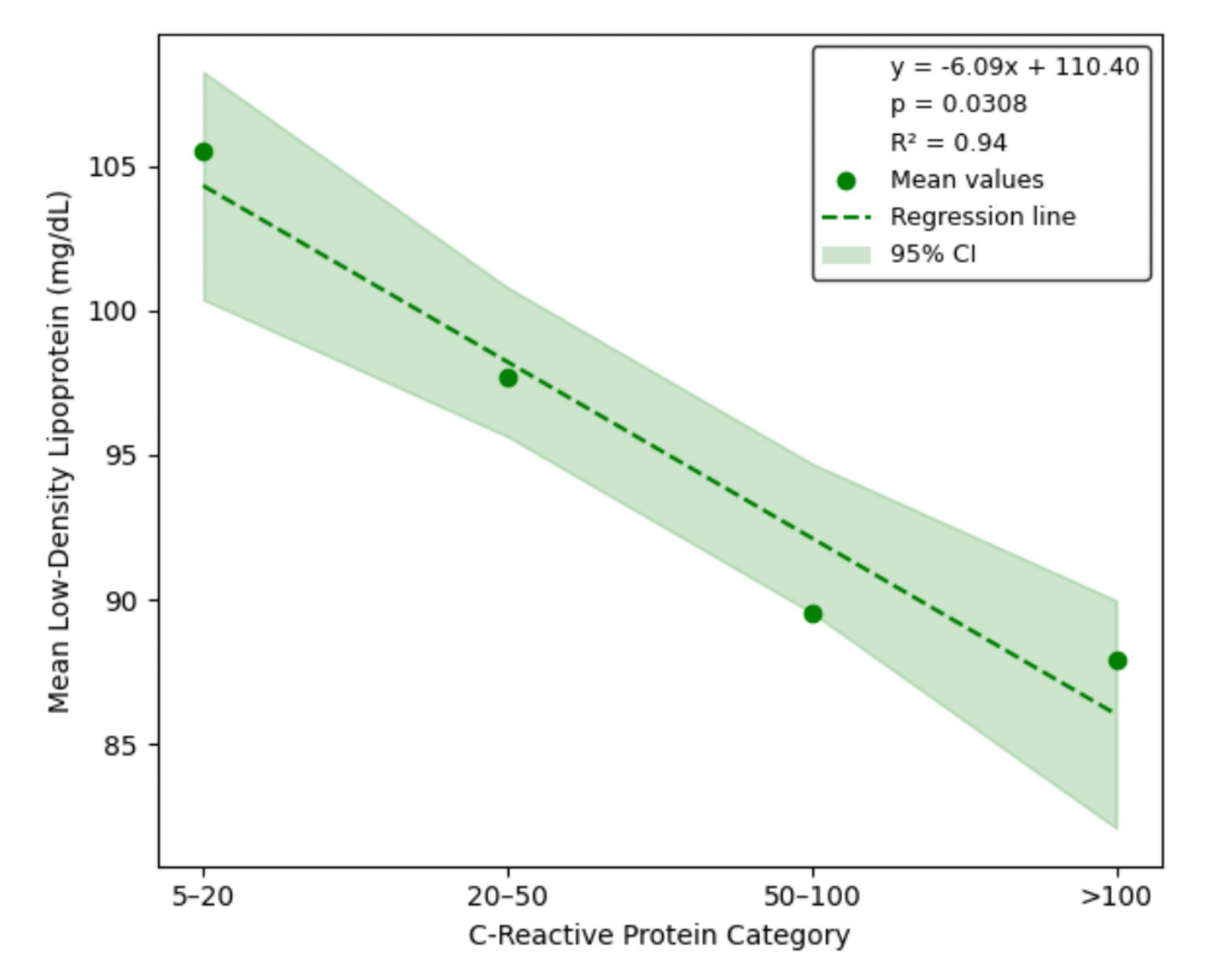
Linear regression of LDL by CRP category The CRP category is inversely associated with LDL levels in hospitalized adults (slope = -6.09 mg/dL per CRP category, 95% CI: -10.79 to -1.38, p = 0.0308, R² = 0.94), reflecting strong and statistically significant LDL suppression in the context of systemic inflammation. CRP, C-reactive protein; LDL, low-density lipoprotein; CI, confidence interval

**Figure 2:**
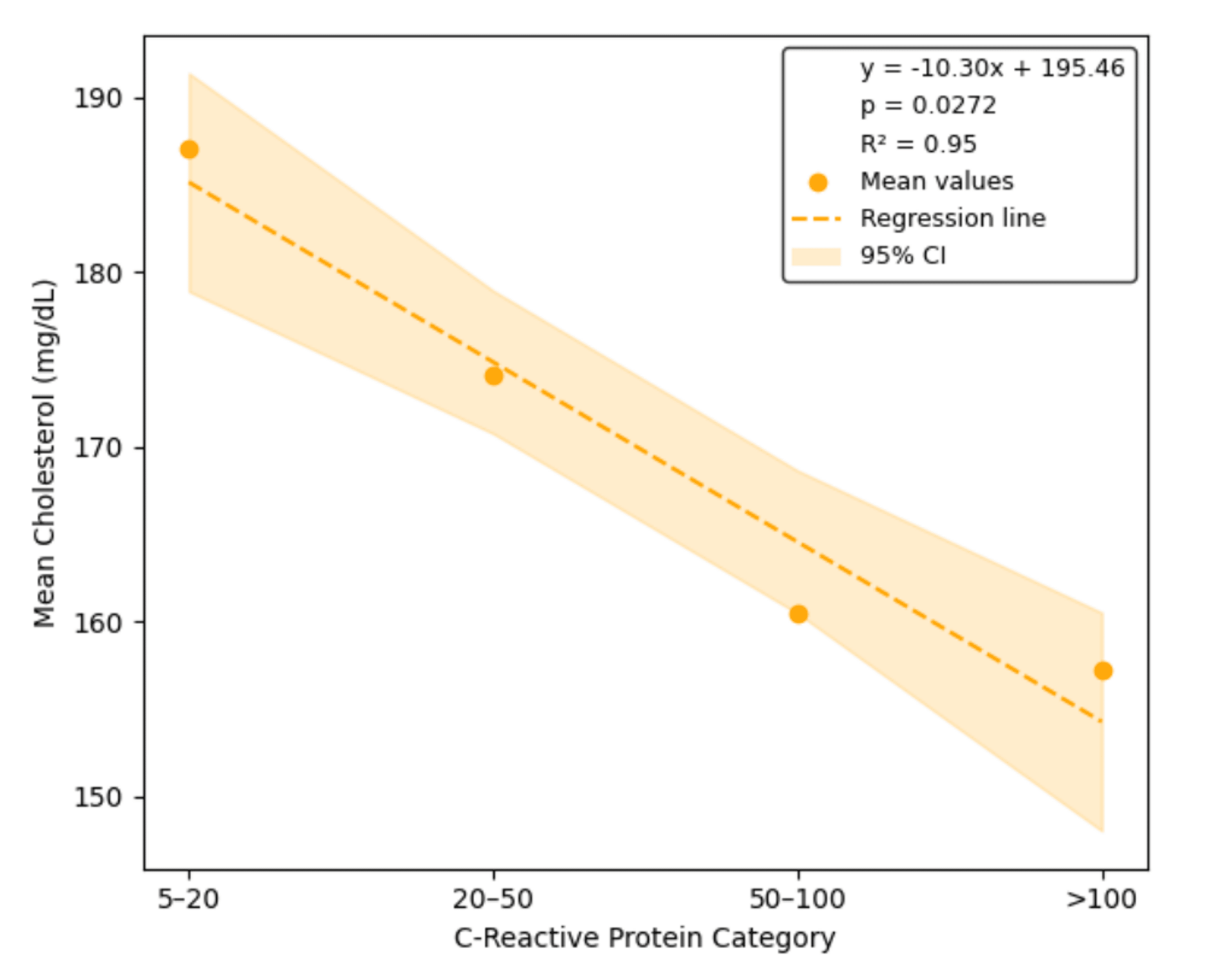
Linear regression of the total cholesterol by CRP category Linear regression demonstrates a statistically significant inverse relationship between CRP category and mean total cholesterol levels (slope = -10.30 mg/dL per CRP category, 95% CI: -17.76 to -2.83, p = 0.0272, R² = 0.95). These findings support a CRP-associated suppression of total cholesterol during systemic inflammation. CRP, C-reactive protein; CI, confidence interval

**Figure 3:**
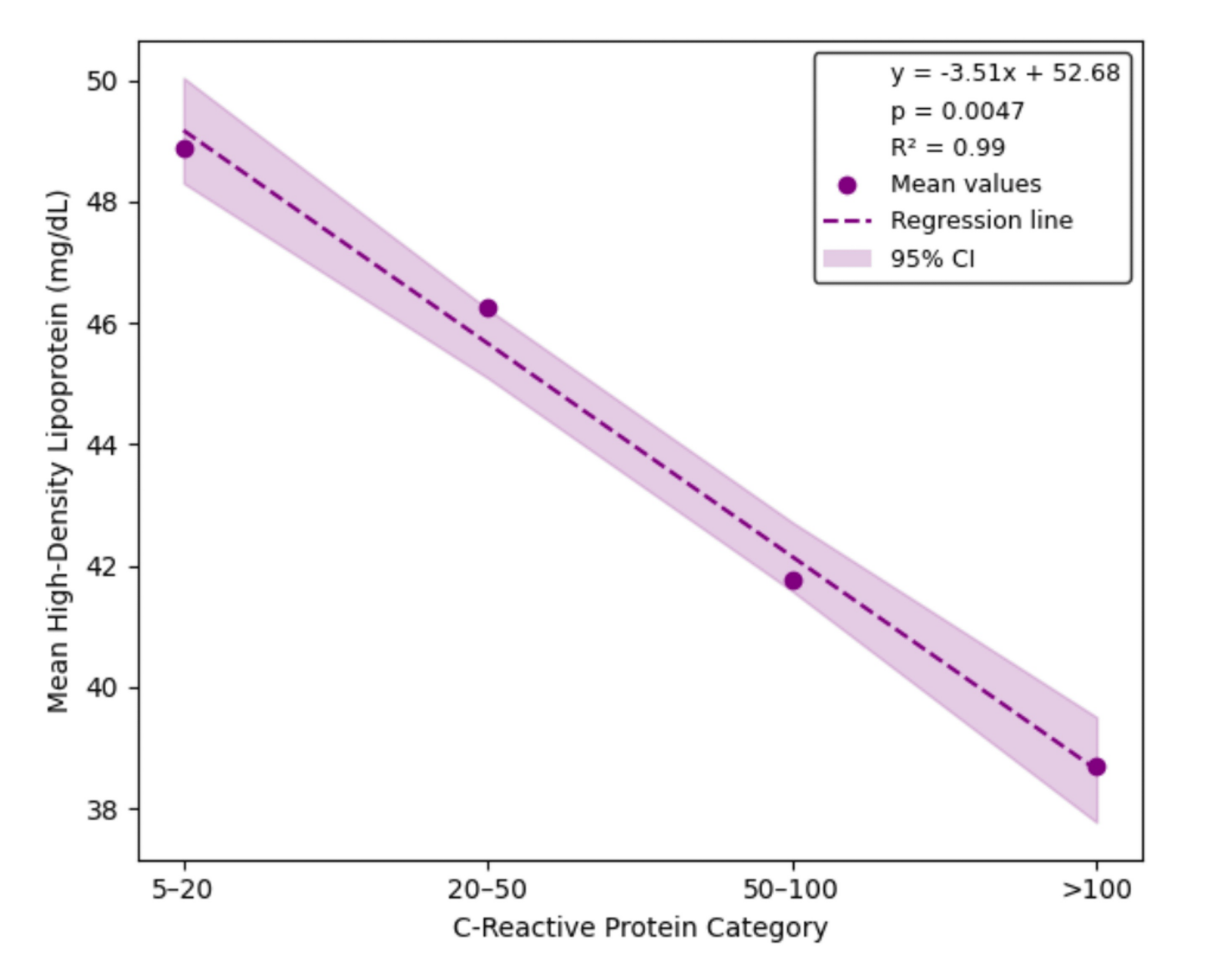
Linear regression of HDL by CRP category Linear regression shows a significant inverse association between CRP category and mean HDL levels (slope = -3.51 mg/dL per CRP category, 95% CI: -4.55 to −2.47, p = 0.0047, R² = 0.99), suggesting inflammation-mediated reduction in HDL across increasing CRP strata. CRP, C-reactive protein; HDL, high-density lipoprotein; CI, confidence interval

**Figure 4:**
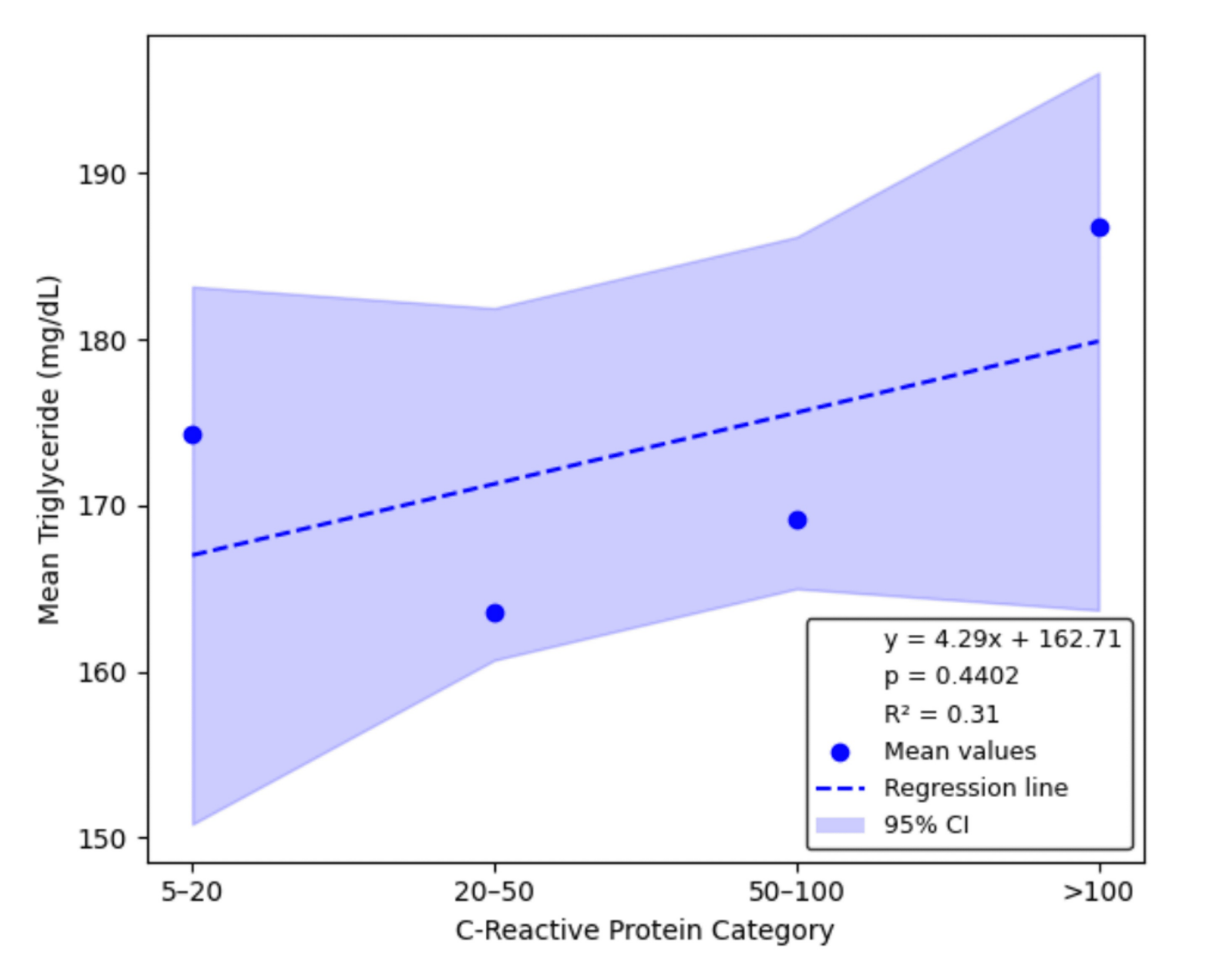
Linear regression of triglycerides by CRP category Triglycerides exhibited a non-significant upward trend across CRP categories (slope = 4.29 mg/dL per CRP category, 95% CI: -15.04 to 23.62, p = 0.4402, R² = 0.31). CRP, C-reactive protein; CI, confidence interval

Linear regression analysis confirmed significant inverse relationships between CRP category and LDL, HDL, and TC, with estimated slopes of -6.09 mg/dL (95% CI: -10.79 to -1.38, p = 0.0308, R² = 0.94), -3.51 mg/dL (95% CI: -4.55 to -2.47, p = 0.0047, R² = 0.99), and -10.30 mg/dL (95% CI: -17.76 to -2.83, p = 0.0272, R² = 0.95), respectively. The slope for TGs was positive (4.29 mg/dL), but the association was not statistically significant (95% CI: -15.04 to 23.62, p = 0.4402, R² = 0.31). These regression results are illustrated in Figures 1-4, each depicting the linear relationship between CRP category and mean lipid level with shaded confidence bands.

## Discussion

Our findings confirm a robust inverse association between CRP levels and lipid parameters, specifically LDL, HDL, and TC, in a diverse inpatient cohort. By demonstrating this relationship outside of autoimmune conditions, we extend the lipid paradox to the broader hospitalized population, suggesting that lipid suppression is a generalizable response to systemic inflammation rather than a disease-specific phenomenon [13,14]. These results provide clinical evidence supporting the mechanistic theory that inflammation suppresses circulating lipid concentrations, which may obscure atherogenic risk during acute illness and paradoxically increase the likelihood of CV events [1,2,5,6].

These findings have important clinical implications. Lipid panels drawn during periods of elevated CRP may underestimate CV risk, potentially delaying the initiation of lipid-lowering therapy [3,4]. This is particularly concerning in hospitalized patients, where inflammation-driven lipid suppression may offer a false sense of cardiometabolic security. To address this, the European League Against Rheumatism recommends applying a 1.5-fold multiplier to CV risk scores in patients with RA to adjust for suppressed lipid values [4]. Our findings suggest that similar adjustments may be appropriate in acutely ill populations, where systemic inflammation can transiently distort lipid profiles and obscure underlying risk.

Beyond masking risk, systemic inflammation may itself exacerbate CV risk. Inflammatory cytokines, such as interleukin-6 (IL-6) and tumor necrosis factor-alpha (TNF-α), contribute directly by suppressing apolipoprotein synthesis, impairing reverse cholesterol transport, and promoting lipoprotein oxidation, all of which drive a shift toward a more atherogenic lipid profile [15,16]. These effects can persist even when LDL levels appear low, contributing to chronic arterial inflammation and increasing the likelihood of CV events [16]. Notably, this inflammatory state can result in dysfunctional HDL and smaller, more oxidized LDL particles, which are key drivers of plaque formation and vascular injury, especially in autoimmune disease contexts such as RA [5]. Additionally, cytokines such as interleukin-1 beta and TNF-α impair cholesterol efflux by downregulating ATP-binding cassette transporter A1 and promoting foam cell formation, thereby exacerbating lipid accumulation and plaque instability [17]. Encouragingly, anti-inflammatory therapies such as methotrexate or prednisone have been shown to partially restore HDL levels and improve lipid transporter activity, highlighting the reversible nature of these inflammation-mediated changes [7,8,18].

Recent population-based studies support this phenomenon: in patients with RA, lower LDL and TC levels have been linked to higher rates of CV events, particularly in those with elevated CRP or erythrocyte sedimentation rate [19-21]. Similar trends have been reported in patients with acute myocardial infarction, where a U-shaped relationship between LDL and mortality was observed only among those with high CRP, suggesting that inflammation modifies the lipid-CVD risk relationship [22]. Taken together, these findings reinforce the clinical relevance of our results, suggesting that inflammation-driven lipid suppression may not only obscure CV risk but also contribute independently to adverse outcomes, highlighting the need for inflammation-adjusted risk stratification in both acute and chronic care settings.

This study has several limitations. First, its retrospective design and reliance on electronic medical records preclude causal inference. Second, we could not account for lipid-lowering medication use, nutritional status, or underlying diagnoses, each of which may independently influence lipid levels. Third, our data lacked granularity on lipid subclasses such as apolipoprotein A-I, apolipoprotein B, and lipoprotein(a), which may provide deeper insights into inflammatory modulation of atherogenic risk. Fourth, there was a temporal limitation in CRP-lipid pairing, as some lipid measurements may have been collected days to months apart from CRP, potentially attenuating observed associations. Fifth, we could not determine fasting status at the time of lipid testing, which may have introduced variability, especially in TG levels. While non-fasting testing is increasingly accepted, this may partly explain the non-linear TG pattern observed. Sixth, we did not stratify patients by primary diagnosis (e.g., sepsis, pneumonia, malignancy), each of which may exert unique effects on lipid metabolism. Seventh, information on glucocorticoid or biologic therapy use was unavailable; these therapies may affect both CRP and lipid metabolism, confounding associations. Finally, as a single-center study, generalizability may be limited. Nonetheless, our large sample size, CRP stratification, and consistent trends support the validity and relevance of our findings.

Future studies should prospectively assess lipid dynamics in relation to inflammation, accounting for medication use, fasting status, and diagnostic subgroups. Longitudinal designs tracking lipid levels before, during, and after acute illness would clarify the duration and clinical implications of transient lipid suppression. Incorporating lipid subclasses and inflammatory cytokines (e.g., IL-6, TNF-α) may further elucidate mechanisms and support inflammation-adjusted CV risk tools. These findings may also inform optimal timing for lipid screening and preventive therapy in hospitalized patients.

## Conclusions

This study demonstrates that elevated CRP levels are significantly associated with lower LDL, HDL, and TC concentrations in hospitalized adults, supporting the existence of the inflammatory lipid paradox beyond chronic autoimmune disease and mechanistic models. These findings highlight the importance of interpreting lipid panels in the context of systemic inflammation, as acute illness may transiently suppress lipoprotein levels and either obscure or amplify true atherogenic risk. Reassessment of lipid profiles after resolution of systemic inflammation may better inform CV risk stratification and guide timely preventive interventions. Further investigation is warranted to determine whether inflammation-induced lipid changes are transient or predictive of long-term CV outcomes.
